# ERK inhibition sensitizes CZ415-induced anti-osteosarcoma activity *in vitro* and *in vivo*

**DOI:** 10.18632/oncotarget.18303

**Published:** 2017-05-30

**Authors:** Gang Yin, Jin Fan, Wei Zhou, Qingfeng Ding, Jun Zhang, Xuan Wu, Pengyu Tang, Hao Zhou, Bowen Wan, Guoyong Yin

**Affiliations:** ^1^ Department of Spine Surgery, The First Affiliated Hospital of Nanjing Medical University, Nanjing, Jiangsu 210029, China; ^2^ Department of Orthopaedics, Changzhou Wujin Hospital Affiliated to Jiangsu University, Changzhou, Jiangsu 213017, China

**Keywords:** osteosarcoma, mTOR, CZ415, ERK, molecular-targeted therapy

## Abstract

mTOR is a valuable oncotarget for osteosarcoma. The anti-osteosarcoma activity by a novel mTOR kinase inhibitor, CZ415, was evaluated. We demonstrated that CZ415 potently inhibited survival and proliferation of known osteosarcoma cell lines (U2OS, MG-63 and SaOs2), and primary human osteosarcoma cells. Further, CZ415 provoked apoptosis and disrupted cell cycle progression in osteosarcoma cells. CZ415 treatment in osteosarcoma cells concurrently blocked mTORC1 and mTORC2 activation. Intriguingly, ERK-MAPK activation could be a major resistance factor of CZ415. ERK inhibition (by MEK162/U0126) or knockdown (by targeted ERK1/2 shRNAs) dramatically sensitized CZ415-induced osteosarcoma cell apoptosis. *In vivo*, CZ415 oral administration efficiently inhibited U2OS tumor growth in mice. Its activity was further potentiated with co-administration of MEK162. Collectively, we demonstrate that ERK inhibition sensitizes CZ415-induced anti-osteosarcoma activity *in vitro* and *in vivo*. CZ415 could be further tested as a promising anti-osteosarcoma agent, alone or in combination of ERK inhibition.

## INTRODUCTION

The explore of new ant-osteosarcoma (OS) agents is extremely important [1–3]. Molecular-targeted therapy has drawn significant attentions for OS treatment [3–5]. Mammalian target of rapamycin (mTOR) is a well-known serine/threonine protein kinase, which is central member in the PI3K-AKT-mTOR cascade [6]. Activation of mTOR could promote a number of pro-cancerous processes, including cell survival, proliferation, and metabolism, as well as angiogenesis, metastases and apoptosis-resistance [6]. Recent studies have confirmed that dysregulation of mTOR signaling in OS [7], which represents a major oncotarget for treatment [8–11].

Two mTOR complexes have been characterized thus far [12–14], including the traditional mTOR complex 1 (mTORC1) and later-discovered mTOR complex 2 (mTORC2) [6]. mTORC1 could be inhibited by rapamycin and its analogs (“rapalogs”), which is composed of mTOR, RAPTOR, PRAS40 and mLST8 [15, 16]. mTORC1 phosphorylates two major downstream proteins, p70S6K1 (S6K1) and eIF4E-binding protein 1 (4E-BP1), promoting various oncogenic behaviors [6, 15, 16]. mTORC2, on the other hand, is assembled by mTOR, Rictor, Sin1 and mLST8, among others [6, 15, 16]. It functions as AKT kinase, which phosphorylates AKT at Ser-473 [15, 16]. It has been shown that both mTORC1 and mTORC2 are over-activated in human OS, which is associated with cancer initiation and progression [7]. Recent research efforts have developed CZ415 as a highly-selective and potent mTOR kinase inhibitor [17]. It blocks both mTORC1 and mTORC2 in cellular model [17]. Its activity against human OS cells was evaluated in the current study.

## RESULTS

### CZ415 is anti-survival and anti-proliferative to human OS cells

**Figure 1:**
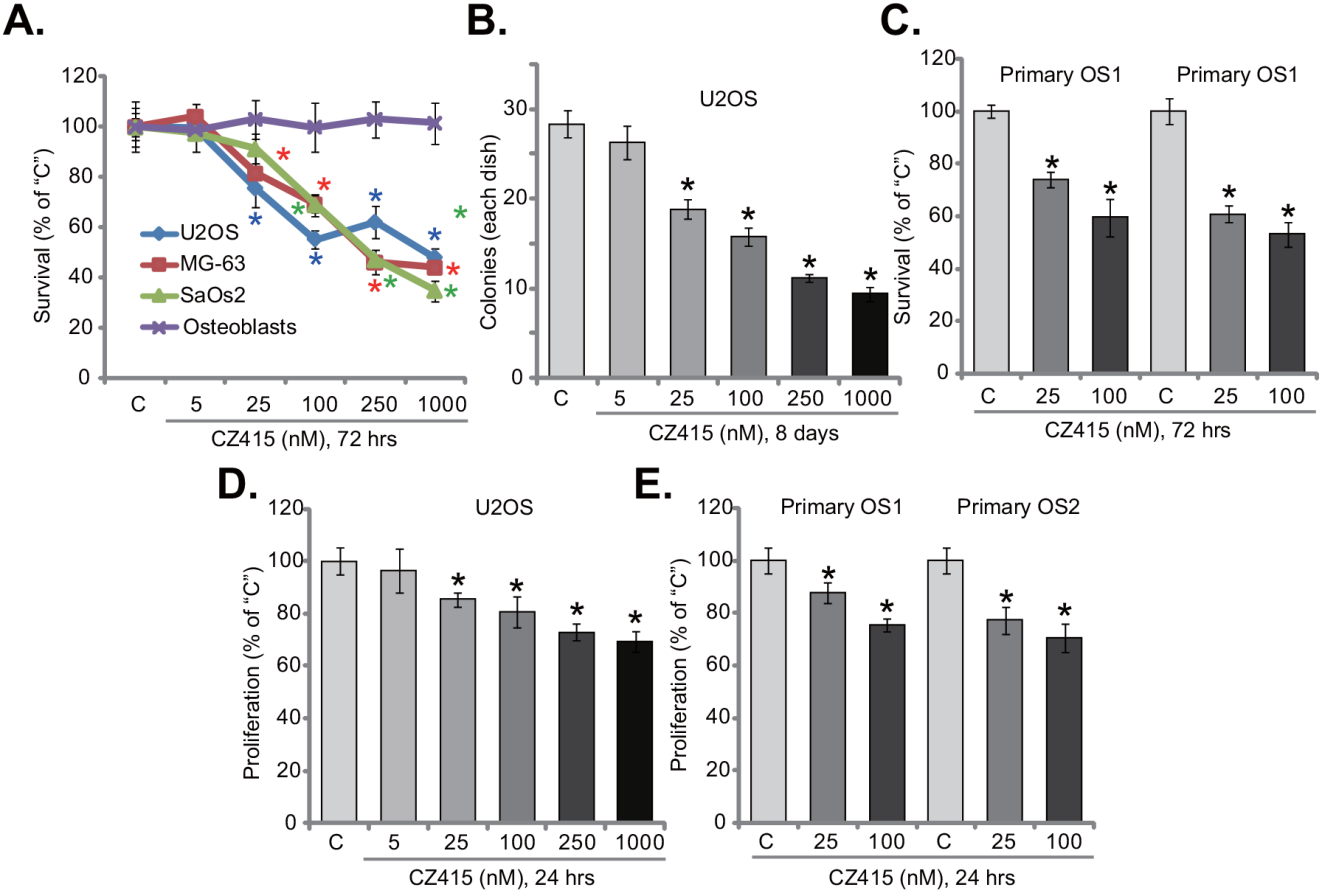
CZ415 is anti-survival and anti-proliferative to human OS cells Established OS cell lines (U2OS, MG-63 and SaOs2), primary murine osteoblasts (“Osteoblasts”), or the primary human OS cells (“primary OS1” and “primary OS2”), were either untreated (“C”, same for all Figures), or treated with designated concentration of CZ415, cells were further cultured for indicated time. Cell survival **(A-C)** and proliferation **(D** and **E)** were tested by listed assays. For each assay, n=5. *p<0.05 *vs.* group “C”. Experiments in this figure were repeated four times, with similar results were obtained.

In this study, we aim to examine the potential activity of CZ415, the novel mTOR kinase inhibitor [17, 18], against human OS cells. Three well-known established OS cell lines, including U2OS, MG-63 and SaOs2, were treated with CZ415. MTT survival assay results in Figure 1A demonstrated that treatment with CZ415 inhibited survival of the OS cell lines. CZ415 displayed a concentration-dependent activity in decreasing OS cell survival (Figure 1A). CZ415 was yet non-cytotoxic to primary murine osteoblasts (Figure 1A), as MTT OD was unchanged before and after CZ415 treatment (Figure 1A). To further confirm the anti-survival activity of CZ415, colony formation assay was performed. Results demonstrated that CZ415, at 25-1000 nM (8 days incubation), significantly decreased the number of viable U2OS colonies (Figure 1B). Next, two primary human (patient-derived) OS cell lines were established: named “primary OS1” and “primary OS2”. The primary OS cells were also treated with CZ415 (25/100 nM). MTT assay results showed that the survival of these primary OS cells was also inhibited following the applied CZ415 treatment (Figure 1C).

Since activation of mTOR is important for cancer cell proliferation [6], the activity of CZ415 on OS cell proliferation was tested next. BrdU incorporation ELISA assay was performed. Results in Figure 1D showed that treatment of CZ415 (at 25-1000 nM) in U2OS cells significantly decreased BrdU ELISA OD, suggesting its anti-proliferative activity. Similarly, in the primary human OS cells (“primary OS1” and “primary OS2”), CZ415 (25/100 nM) largely inhibited BrdU incorporation (Figure 1E). Notably, for the BrdU assay, OS cells were treated with CZ415 for only 24 hours, when no significant survival reduction/cell death was noticed. Collectively, these results suggest that CZ415 is anti-survival and anti-proliferative to human OS cells.

### CZ415 provokes apoptosis in OS cells

**Figure 2:**
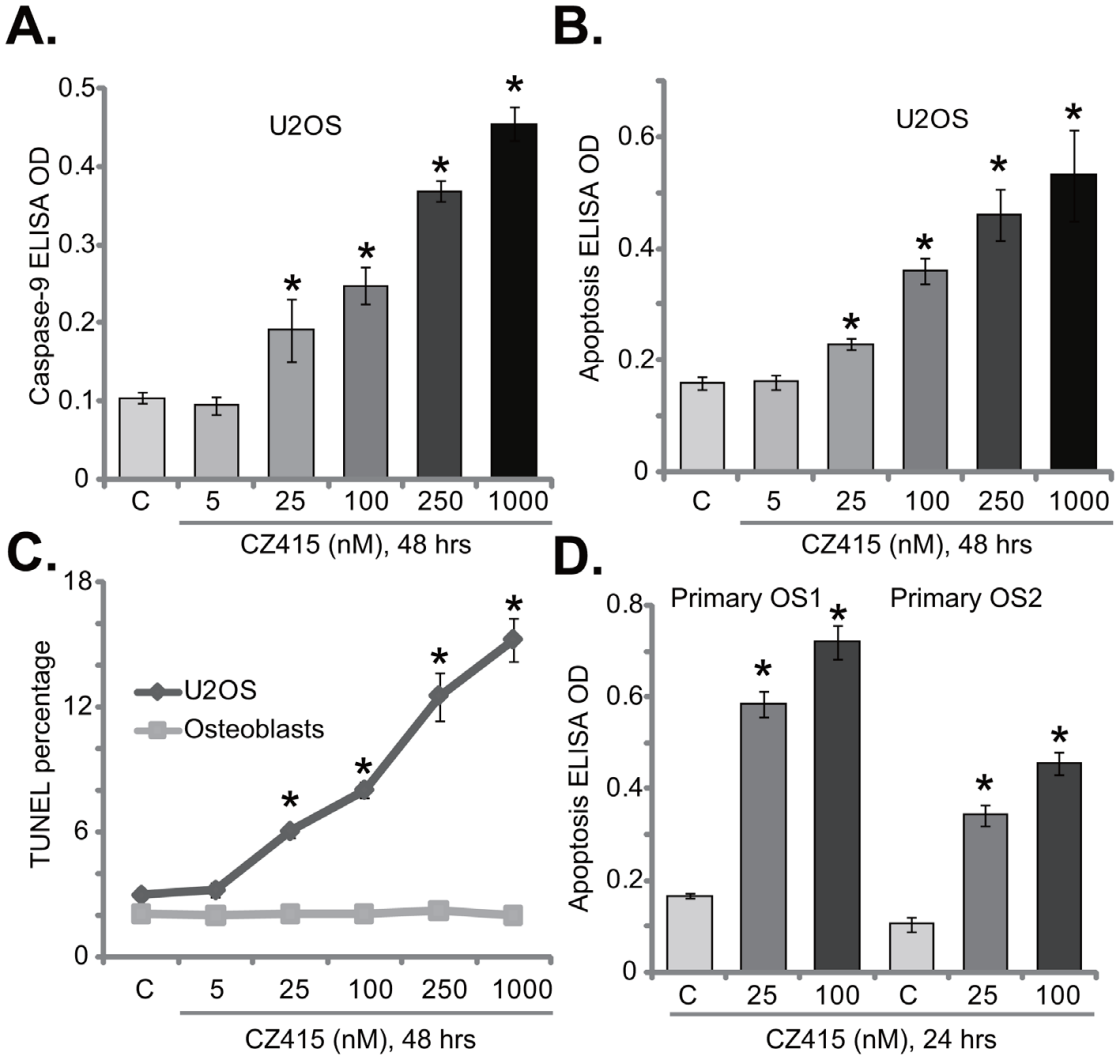
CZ415 provokes apoptosis in OS cells U2OS cells **(A-C)**, primary murine osteoblasts (“Osteoblasts”, **C**), or the primary human OS cells (“primary OS1” and “primary OS2”) **(D)** were treated with designated concentration of CZ415, cells were further cultured for indicated time. Cell apoptosis was tested by listed assays. For each assay, n=5. *p<0.05 *vs.* group “C”. Experiments in this figure were repeated five times, with similar results were obtained.

Next, we tested the potential activity of CZ415 on OS cell apoptosis. Caspase-9 activity assay results in Figure 2A demonstrated that CZ415 concentration-dependently activated caspase-9 in U2OS cells. Meanwhile, Histone DNA apoptosis ELISA OD was increased following CZ415 (at 25-1000 nM) treatment in U2OS cells (Figure 2B). Further, the percentage of U2OS cells with TUNEL positive nuclei was also significantly elevated with CZ415 (at 25-1000 nM) treatment (Figure 2C). These results confirm that CZ415 induced apoptosis in U2OS cells (Figure 2A-2C). On the other hand, same CZ415 treatment failed to induce significant apoptosis in primary osteoblasts (Figure 2C), confirming selective activity of CZ415 against cancerous cells. The pro-apoptosis activity of CZ415 was also observed when added to primary OS cells (“primary OS1” and “primary OS2”), where CZ415 (at 25-1000 nM) treatment significantly increased Histone DNA apoptosis ELISA OD (Figure 2D). Collectively, these results confirm that CZ415 provokes apoptosis in OS cells.

### CZ415 disrupts OS cell cycle progression, causing G1-S arrest

**Figure 3:**
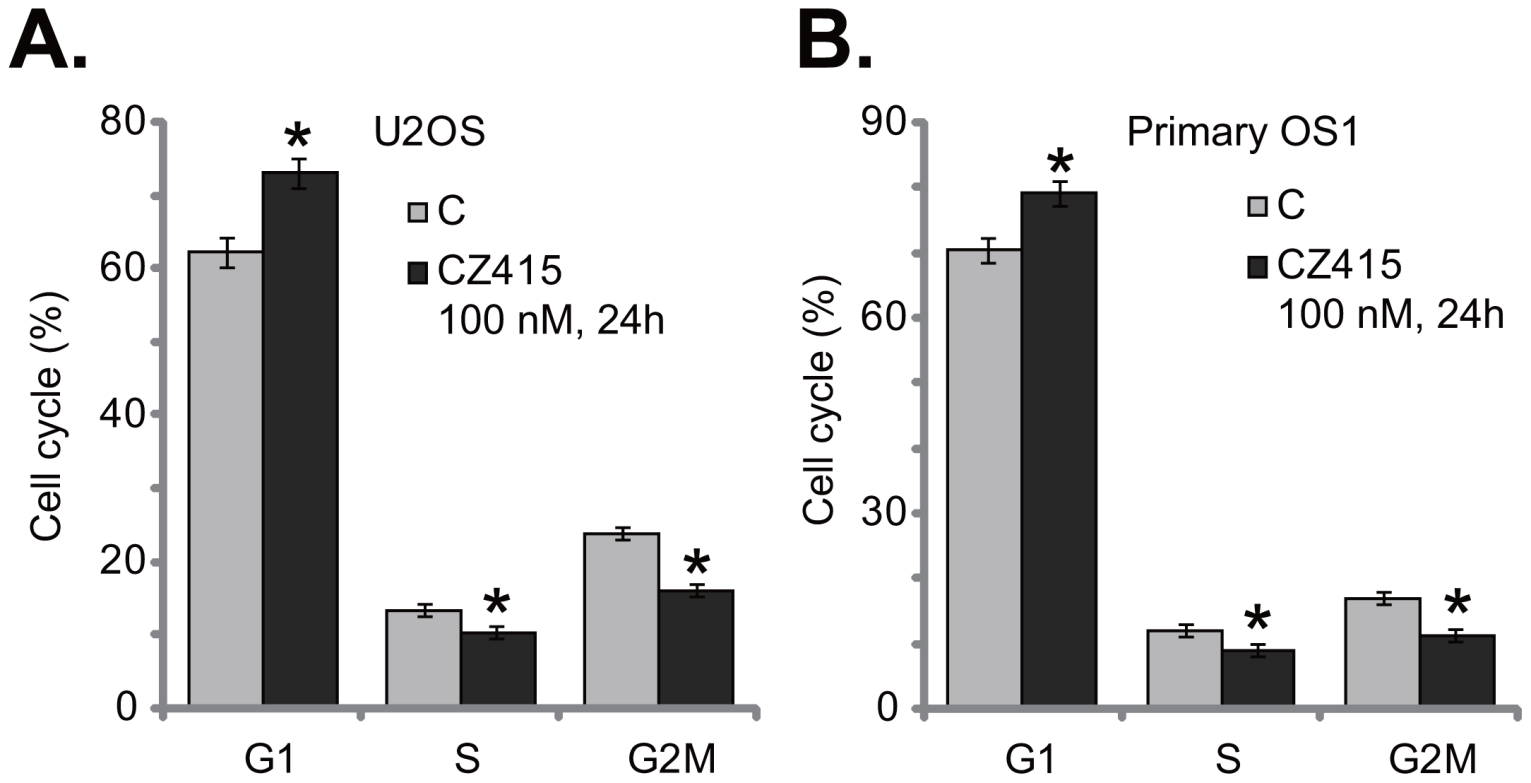
CZ415 disrupts OS cell cycle progression, causing G1-S arrest U2OS cells **(A)** or the primary human OS cells (“primary OS1”) **(B)** were treated with CZ415 (100 nM) for 24 hours, cell cycle was analyzed by PI-FACS assay, and results were quantified. For each assay, n=3. *p<0.05 *vs.* group “C”. Experiments in this figure were repeated three times, with similar results were obtained.

Activation of mTOR is vital for cancer cell cycle progression [6]. Several cell cycle proteins, including Cyclin D1 and Cyclin E, were mTOR-dependent [6]. Thus, the potential activity of CZ415 on cell cycle progression was tested. Quantified results in Figure 3A showed that treatment with CZ415 (100 nM for 24 hours) in U2OS cells led to increase of G1 phase, but significant reduction of S and G2M phases. These results imply that CZ415 possibly induced G1-S arrest in U2OS cells (Figure 3A). Similarly in the primary OS cells, G1 phase increase and S/G2M phase decrease were observed after CZ415 (100 nM for 24 hours) treatment (Figure 3B). Therefore, CZ415 disrupts OS cell cycle progression, causing G1-S arrest to favor proliferation inhibition.

### CZ415 blocks mTORC1 and mTORC2 activation in OS cells

**Figure 4:**
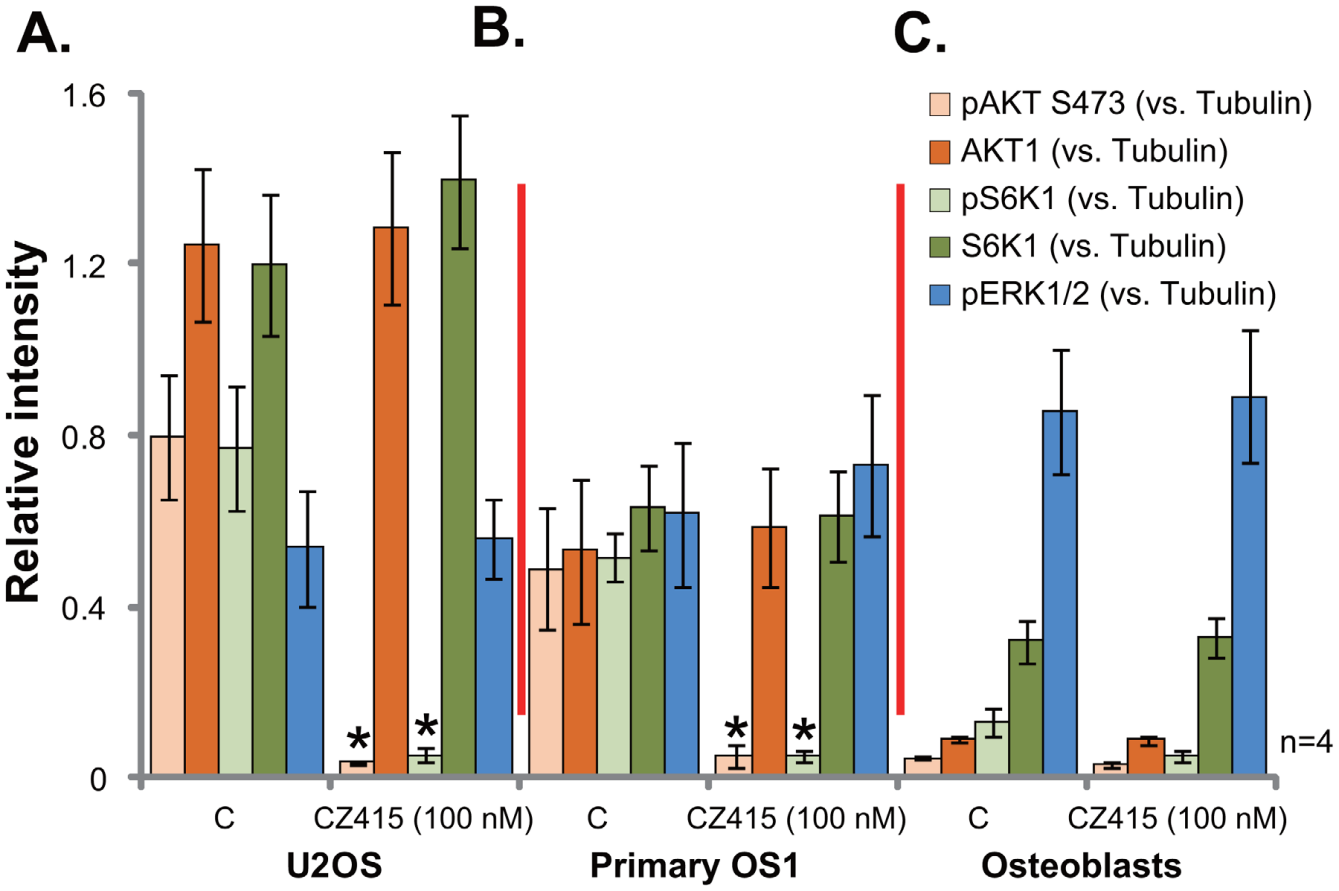
CZ415 blocks mTORC1 and mTORC2 activation in OS cells U2OS cells **(A)**, the primary human OS cells (“primary OS1”) **(B)** or primary murine osteoblasts (“Osteoblasts”) **(C)** were treated with/out CZ415 (100 nM) for 3 hours, expressions of the listed proteins were shown. Four sets of blot data were quantified. *p<0.05 *vs.* group “C”.

Since CZ415 is a newly-developed mTOR kinase inhibitor [17, 18], it presumably should block mTORC1 and mTORC2 activation. Indeed, in the U2OS cells, treatment of CZ415 (100 nM, 3 hours) blocked p-S6K1 (Thr-389, the indicator of mTORC1 activation) and p-AKT (Ser-473, the indicator of mTORC2 activation) [6] (Four sets of blot data were quantified in Figure 4A). ERK-MAPK activation, tested by p-ERK1/2, was not affected by the same CZ415 treatment (Figure 4A). Similar results were also achieved in the primary human OS cells (“Primary OS1”), where CZ415 (100 nM, 3 hours) almost blocked activation of mTORC1 (p-S6K1) and mTORC2 (p-AKT, Ser-473), but not ERK (Four sets of blot data were quantified in Figure 4B). On the other hand, in the primary osteoblasts, basal activation and expression of AKT-S6K1 were much lower than those in the OS cells (Four sets of blot data were quantified in Figure 4C), which could be the primary reason of ineffectiveness of CZ415 in these non-cancerous cells (Figures 1 and 2). Collectively, these results demonstrate that CZ415 blocks mTORC1 and mTORC2 activation in OS cells.

### ERK activation is a primary resistance factor of CZ415 in OS cells

**Figure 5:**
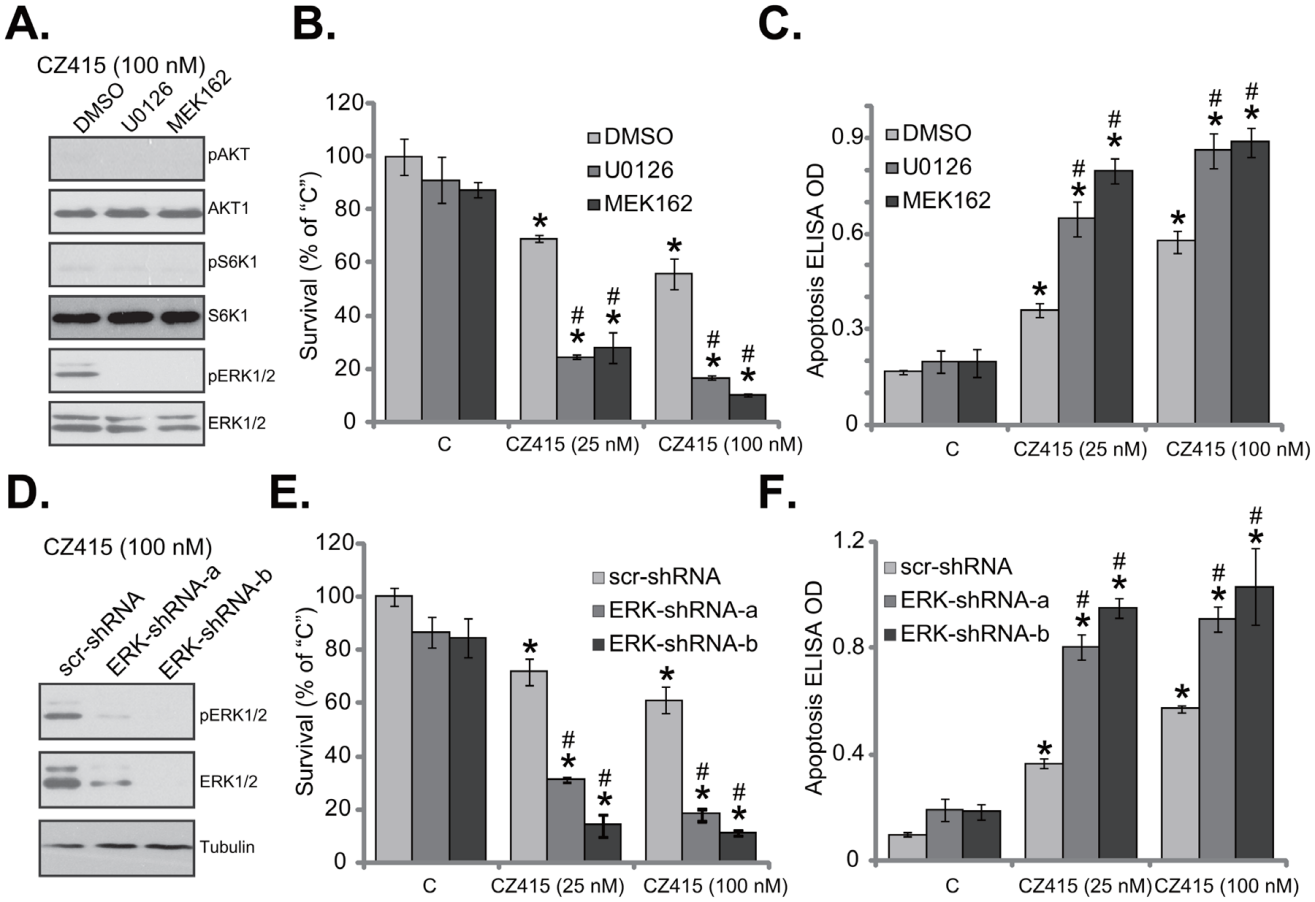
ERK activation is a primary resistance factor of CZ415 in OS cells U2OS cells were treated with CZ415 (25/100 nM), or plus MEK-ERK inhibitor U0126 (1 μM) or MEK162 (1 μM), expressions of listed proteins were tested **(A)**. Cell survival (**B**, MTT assay, 72 hours) and apoptosis (**C**, Histone DNA ELISA assay, 48 hours) were also examined. U2OS cells, expressing scramble control shRNA (“scr-shRNA”) or listed ERK shRNA (“ERK-shRNA-a/-b”), were treated with CZ415 (25/100 nM), expressions of listed proteins were shown **(D)**. Cell survival **(E)** and apoptosis **(F)** were tested similarly. “DMSO” stands for 0.1 % of DMSO **(B** and **C)**. For each assay, n=5. *p<0.05 *vs.* group “C”. ^#^ *p*<0.05 *vs.* CZ415 only treatment **(B** and **C)**. ^#^ *p*<0.05 *vs.* “scr-shRNA” group **(E** and **F)**. Experiments in this figure were repeated three times, with similar results were obtained.

The above results demonstrated that CZ415 blocked mTORC1/2, but not ERK, in human OS cells. ERK-MAPK is a well-established oncogenic signaling in OS [2, 3], whether ERK inhibition could affect CZ415’s activity in OS cells was tested next. First, MEK-ERK inhibitors, including U0126 [19, 20] and MEK162 [21, 22] were applied. Expectably, U0126 and MEK162 blocked ERK activation (p-ERK1/2) in CZ415-treated U2OS cells (Figure 5A). The MEK-ERK inhibitors didn’t affect CZ415-induced mTORC1/2 inactivation (Figure 5A). Remarkably, U0126 or MEK162 significantly potentiated CZ415-induced survival reduction (Figure 5B) and apoptosis (Figure 5C) in U2OS cells. In another words, CZ415’s sensitivity against U2OS cells was increased following MEK-ERK inhibition (Figure 5B and 5C).

To exclude the possible off-target effects of the MEK-ERK inhibitors, shRNA strategy was utilized to silence ERK. Two non-overlapping ERK1/2 shRNAs were applied (“ERK-shRNA-a/-b”), both efficiently downregulated ERK1/2 in U2OS cells (Figure 5D). Consequently, ERK activation, or p-ERK1/2 was also largely inhibited (Figure 5D). ERK silence largely potentiated CZ415-induced cytotoxicity in U2OS cells, leading profound viability reduction (Figure 5E) and apoptosis (Figure 5F). Notably, ERK inhibition or silence alone only induced minor cytotoxicity in U2OS cells (Figure 5B, 5C, 5E, 5F). Collectively, these results suggest that ERK could be a primary resistance factor of CZ415, and ERK inhibition/silence could largely increase CZ415’s sensitivity in OS cells.

### The anti-OS activity of CZ415 *in vivo*

**Figure 6:**
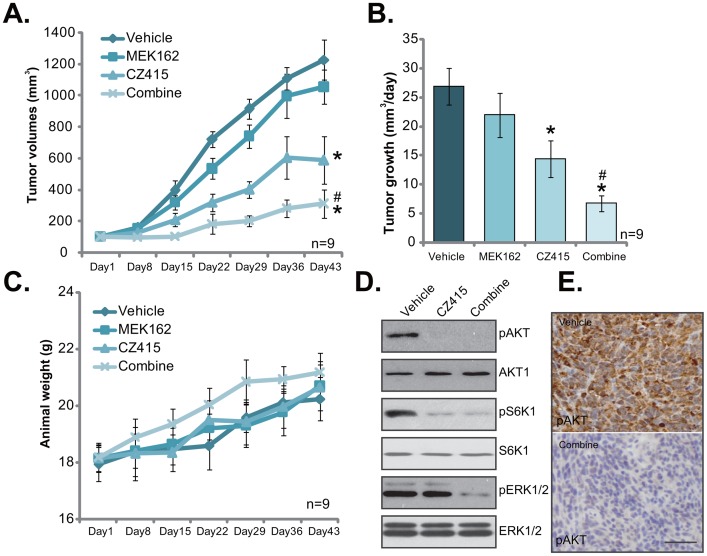
The anti-OS activity of CZ415 *in vivo* U2OS tumor-bearing SCID mice were administrated (by gavage) daily with vehicle control (Saline, “Veh”), CZ415 (25 mg/kg) and/or MEK162 (5 mg/kg) for 21 days (10 mice per group). Tumor volumes **(A)** and mice body weights **(C)** were recorded every weekly for 6 weeks. Estimated tumor growth (mm^3^ per day) was also presented **(B)**. At day-3, one tumor of each group was separated, and expressions of listed proteins in tumor lysates were tested by Western blot assay (**D**, tumor lysates). IHC staining assay was performed to test p-AKT Ser-473 in tumor slide (**E**, bar=100 μm). * *p* <0.05 *vs.* “Vehicle” group. ^#^ *p* <0.05 *vs.* “CZ415” only group.

At last, we tested the potential anti-OS activity of CZ415 *in vivo*. A significant number of U2OS cells were injected *s.c*. to the SCID (severe combined immuno-deficient) mice, and xenograft OS tumors were established. Tumor growth curve was recorded. Results in Figure 6A demonstrated that CZ415 administration (25 mg/kg body weight [17], gavage, daily for 21 days) in SCID mice significantly inhibited U2OS tumor growth. Remarkably, co-administration of MEK162 (5 mg/kg, gavage, again daily for 21 days) [8] dramatically potentiated CZ415-induced anti-OS activity *in vivo*, leading to profound inhibition of U2OS tumors (Figure 6A). Estimated daily tumor growth results in Figure 6B demonstrated that CZ415 and MEK162 co-administration led to dramatic inhibition of U2OS tumor growth, showing lowest daily tumor growth (Figure 6B). The co-administration was dramatically more potent than CZ415 single treatment in inhibiting U2OS tumors (Figure 6B). Mice body weights, on the other hand, were not significantly affected by the single or combined treatments (Figure 6C). Therefore, the treatment regimens were relatively safe to the tested SCID mice.

At day-3, one tumor per each group was separated, and expressions of listed proteins in tumor lysates were tested. In line with *in vitro* findings, CZ415 plus MEK162 co-administration led to in-activation of mTORC1/2 (p-S6K1/p-AKT Ser473) and ERK (p-ERK1/2) in U2OS tumor tissues (Figure 6D). Signal treatment only led to inhibition of each single pathway (Figure 6D). IHC images further confirmed p-AKT Ser473 inhibition in the co-administrated-tumor tissues (Figure 6E). Together, these results demonstrated that CZ415 oral administration inhibited U2OS tumor growth in SCID mice. Its activity was further potentiated with co-administration of MEK162.

## DISCUSSION

OS still is common and lethal primary bone tumor, with an age-adjusted incidence of 4.4 new cases per million each year [3, 23], causing large mortalities among children [3, 23]. For the advanced OS, cancer cells with rapid proliferation potential and high metastatic ability often lead to poor prognosis [2, 3, 23–25]. Further, chemo-resistance will also be developed following conventional chemotherapy treatment. Thus, molecular-targeted therapy is the current research focus of OS [2, 3, 23–25]. The search for novel anti-OS agents is extremely important [3, 23]. Aberrations of mTOR pathway have often observed in OS, due to for example PTEN deletion and PIK3CA mutations [7]. These evidences suggest an opportunity for focused therapeutic strategies against mTOR in OS.

Traditional mTORC1 inhibitors, *i.e*. rapamycin and rapalogs (RAD001, CCI-779, AP23573), have been tested in preclinical OS studies [26, 27]. For example, a recent report found that those receiving rapamycin compared to a group of tyrosine kinase inhibitors had a better progression-free survival (PFS), although the difference in median PFS was modest [28]. RAD001 has demonstrated activity against OS in a pediatric phase I study, some OS patients experienced prolonged stable disease [29]. Yet, using of these mTORC1 inhibitors only could induce weak to moderate anti-tumor activity [30, 31], possibly due to the following reasons. Rapalogs directly binds to FKBP12, leading to incompletion inhibition of 4E-BP1 phosphorylation and mTORC1 [30, 31]. Further, rapalogs had limited effect on mTORC2, which is also important for OS progression [30, 31]. More importantly, following mTORC1 inhibition, feedback activation of several key pro-cancerous signalings, *i.e*. AKT and ERK-MAPK, could significantly inhibit the activity of rapalogs [30, 31]. Not to mention the solubility of mTORC1 inhibitors is also not satisfactory [15, 32]. Therefore, mTOR kinase inhibitors, blocking both mTOR1 and mTORC2 activation, are being developed [33, 34].

CZ415 is a novel mTOR kinase inhibitor, which has a decent K_d_ (nM ranges) and much improved pharmacokinetic/pharmacodynamic properties [17, 18]. It has a extremely high affinity and selectivity for mTOR [17, 18]. It inhibits mTOR kinase activity, thus blocking both mTORC1 and mTORC2 simantanuously [17, 18]. In the current study, we show that CZ415 blocked mTORC1 and mTORC2 activation, and potently inhibited OS cell survival and proliferation. More importantly, oral administration of CZ415 at well-tolerated dose significantly inhibited U2OS tumor growth in SCID mice. Therefore, CZ415 could be further tested as a promising anti-OS agent.

One novel finding of this study is that ERK activation could be the major resistance factor of CZ415 in OS cells. Concurrent activation of multiple oncogenic cascades is a characteristic marker of OS [2, 3, 23–25]. Inhibition of single pathways, in our case mTOR blockage by CZ415, could therefore only lead to moderate anti-OS activity [2, 3, 23–25]. Simultaneous inhibition of ERK-MAPK signaling, via pharmacological or genetic methods, could then lead to much improved anti-OS activity. As a matter of fact, ERK-MAPK inhibition could be a fine strategy to sensitize mTOR kinase inhibitors in preclinical cancer studies [35, 36]. It would be interesting to possibly test the combination strategy in clinical OS studies.

## MATERIALS AND METHODS

### Chemicals and reagents

CZ415, MEK162 and U0126 were purchased from MCE China (Beijing, China). The antibodies of the current study were provided by Cell Signaling Tech (Denver MA). The enhanced chemiluminescence (ECL) reagents were purchased from Pierce (Rockford, IL). The cell culture reagents were purchased from Gibco (Suzhou, Jiangsu, China).

### Culture of OS cell lines

Human osteosarcoma cell lines, U2OS, MG-63 and SaOs2, were purchased from the IBS cell bank of Fudan University (Shanghai, China). Cells were cultured in routine DMEM/MEM with FBS medium [37, 38]. DNA fingerprinting and profiling were performed to verify the cell lines origin, and to distinguish them from cross-contamination. Population doubling time, colony forming efficiency, and morphology under phase contrast were also measured every four months to confirm the phonotype of cell lines.

### Primary culture of murine osteoblasts

Primary culture of murine osteoblasts was described in our previous study [39]. Briefly, the murine calvariae were bathed in α-MEM. The trimmed calvariae were washed, and were subjected collagenase-I (Sigma) digestion. Digestions 3-5 were neutralized with α-MEM medium, pooled, and filtered. The single cell suspension was resuspended in 3-5 mL α-MEM plus 20% FBS. Cells were counted and cultured until reaching confluence, half of the medium was renewed every two days.

### Primary culture of human OS cells

As described previously [12, 37], surgery-isolated osteoblastoma tissues (from two independent patients) were washed and minced into small pieces, followed by mechanically disassociating. Tissues were then digested via collagenase I (Sigma). Digestions 3-5 were neutralized, pooled, and filtered. Single cell suspensions of primary OS cells were re-suspended in described complete medium [40]. The protocols using primary human specimen were in accordance with the principles expressed in the Declaration of Helsinki, and were approved by the institutional review board (IRB) and Ethics Board of authors’ institutions. The two participating patients (male, 13 and 14 years old, administrated at authors institution) each provided written-informed consent.

### MTT assay

Briefly, cells were plated at 6 × 10^3^ cells/well onto 96-well plates. After the applied treatment, MTT (Sigma) assay was applied to test cell survival. The detailed protocol is described in our previous study [39].

### Colony formation assay

Cells with applied CZ415 treatment were plated onto 6-well plates at 2 × 10^4^ cells per well. CZ415-containg medium was switched every two days for a total of 8 days. The remaining proliferative colonies were fixed, and manually counted.

### Cell cycle analysis

Cells with applied CZ415 treatment were fixed with 70% ethanol, which were then stained with propidium iodide (PI). Cells were then subjected to FACS analysis on a Beckman Coulter flow cytometer. Cell cycle distribution was recorded.

### BrdU ELISA assay

BrdU ELISA assay kit, purchased from Cell Signaling Tech (Nanjing, China) was applied to quantify cell proliferation [41]. The ELISA OD value of treatment group (at 405 nm) was always normalized to control group.

### TUNEL staining assay

Cell apoptosis was examined by the TUNEL [terminal dexynucleotidyl transferase(TdT)-mediated dUTP nick end labeling] staining assay [42]. The percentage of TUNEL positive cells was calculated under a fluorescent microscope from at least 100 cells per treatment in five independent experiments.

### Histone DNA apoptosis ELISA assay

DNA fragmentation was examined by the Histone DNA enzyme-linked immunosorbent assay (ELISA) kit using the photometric sandwich immunoassay of cytoplasmic histone-associated DNA fragments (Roche, Shanghai, China). The detailed protocol was described previously [39].

### Caspase-9 activity assay

Caspase-9 activity was determined via the Apo-ONE homogeneous caspase-9 activity kit (Promega, Shanghai, China). The caspase-9 substrate Rhodamine 110, or (Z-LEHD-R110), exists as a pro-fluorescent substrate prior to the assay. Addition of active caspase-9 will result in the cleavage of the LEHD peptides and following excitation at 500 nm. Lysate samples and substrates were incubated at 37 °C for 60 min, and then analyzed in a fluorescent spectrophotometer at 500 nm. Relative fluorescent intensity (in optic density, OD) was recorded as the indicator of the caspase-9 activity.

### Western blotting assay

Cells or tumor tissues were washed and resuspended in described tissue lysis buffer [39, 43]. Protein lysates (50 μg per sample) were separated by sodium dodecyl sulfate-polyacrylamide gel electrophoresis (SDS-PAGE) with 10 % polyacrylamide gel, and were transferred onto PVDF membrane (Millipore). Afterwards, the membrane was blocked, followed by incubation with specific primary and secondary (HRP-conjugated) antibodies. Antigen-antibody complex was detected by enhanced chemiluminescence (ECL) reagents [44–46].

### ERK1/2 shRNA knockdown

The two distinct lentiviral ERK1/2 shRNAs, with non-overlapping shRNA sequences, were provided by Dr. Chen at Fudan University (Shanghai, China). The scramble control shRNA were purchased from Santa Cruz Biotech (sc-108065). All sequences were verified by commercial sequencing (Suzhou Jikai). OS cells were infected with lentiviral shRNA at a multiplicity of infection (MOI) of 10. Afterwards, cells were maintained with puromycin (0.5 μg/mL) to establish stable cells. ERK1/2 knockdown in stable cells was verified by Western blotting assay.

### Mice U2OS xenograft assay and immunohistochemistry (IHC) staining

As described [12], CB.17 female SCID (severe combined immuno-deficient) mice, weighted 17.0-18.2 g, were maintained at the Animal Facility of Soochow University (Suzhou, China). For each mouse, 3 × 10^6^ U2OS cells were inoculated into the right flanks via subcutaneously (*s.c*.) injection. When the xenografts were about 100 mm^3^ in volume, mice were randomly into four groups, and were treated as described. Mice body weight and bi-dimensional tumor measurements were recorded every 7 days for a total of 42 days. Tumor volume was estimated using the standard formula: (length × width^2^)/2. All animal protocols were approved by IACUC of Nanjing Medical University. Tumor tissues were also subjected to IHC staining. IHC of U2OS xenografts was performed using the same protocol as described [47].

### Statistical analysis

The quantitative data presented in this study was mean ± standard deviation (SD). Statistical differences were analyzed by one-way ANOVA with post hoc Bonferroni test.

## CONCLUSIONS

Collectively, we demonstrate that ERK inhibition sensitizes CZ415-induced anti-OS activity *in vitro* and *in vivo*. CZ415 could be further tested as a promising anti-OS agent, alone or in combination of ERK inhibition.
